# Availability and feasibility of structured, routine collection of comorbidity data in a colorectal cancer multi-disciplinary team (MDT) setting

**DOI:** 10.1007/s00384-018-3062-2

**Published:** 2018-05-03

**Authors:** A. A. Abukar, A. Ramsanahie, K. Martin-Lumbard, E. R. Herrington, V. Winslow, S. Wong, S. Ahmed, M. A. Thaha

**Affiliations:** 1 Blizard Institute, National Bowel Research Centre, Barts and The London School of Medicine and Dentistry, Queen Mary University London, London, UK; 2 Department of Colorectal Surgery, Surgery & Cancer CAG, The Royal London Hospital, Barts Health NHS Trust, London, UK

**Keywords:** Colorectal cancer, MDT, Comorbidity, ACE-27

## Abstract

**Purpose:**

Availability of comorbidity assessment at multi-disciplinary team (MDT) discussions is cornerstone in making the MDT process more robust and decisive in optimising treatment and improving quality of survivorship. Comorbidity assessments using tools, such as the ACE-27 questionnaire would aid in optimising the decision-making process at MDTs so that treatment decisions can be made without delay. This study determined the availability of comorbidity data in a CRC MDT and the feasibility of routine comorbidity data collection using the validated ACE-27 questionnaire. Secondary aims determined the optimal time and method of collecting comorbidity data.

**Methods:**

A retrospective mapping exercise (phase I; 6-months) examined the availability of comorbidity data within the MDT. Phase II prospectively collected comorbidity data using ACE-27 for a 3-month period following a short pilot.

**Results:**

In phase I, 73/135 (54%) patients had comorbidity data readily available informing the MDT discussion; 62 patients lacked this information. After a review of the patient records, it was clear that 41 of these 62 also had comorbidities and 21 out of the 135 had ≥ 2 major system disorders. Common referral sources to the MDT were surgical outpatient clinics (42%) and the endoscopy unit (13%). The average lead-time from referral to MDT discussion was 14 days. In phase II, an ACE-27 questionnaire was prospectively administered in 50 patients, mean age 54 years (range 20–84). Male: female ratio 26:24. Average time to administer ACE-27 was 4.8 min (range 1–15).

**Conclusions:**

The phase I study confirmed the widely acknowledged view of poor comorbidity data availability within a CRC MDT. Phase II demonstrated the feasibility of routinely collecting comorbidity data using ACE-27.

## Introduction

### Comorbidities

Colorectal cancer (CRC) is the second most common cause of cancer death in the UK and the fourth most common type of cancer in men and women. More than 41,000 new cases of CRC were diagnosed in 2014, with an incidence rate of approximately 64 CRC patients per 100,000 individuals [1, 2]. With an ageing population, the prevalence of comorbidities is set to increase [3, 4]. The presence of comorbid conditions affects treatment pathways and prognosis of patients [5]. Comorbidity reduces the quality of life, may complicate major surgical procedures and increases the risk of hospitalisation and mortality [3, 4]. This highlights the need for comorbidity measurements from a clinical point of view as they may help healthcare providers to respond effectively to the overall severity and healthcare needs of the individual, improving the quality of survivorship. Multi-disciplinary team (MDT) meetings are the gold standard in the management of cancer patients in the UK and are similar to tumour boards in other national settings.

### MDT meetings

Currently, all patients diagnosed with or suspected to have cancer are discussed at MDT meetings. MDT meetings usually take place once weekly bringing together healthcare professionals with an array of skills and expertise. They aim to ensure high-quality decision-making with regard to the diagnosis, treatment and aftercare of patients and they are associated with an improved 5-year survival in colorectal cancer [6, 7]. A number of patient-based factors are discussed at MDTs, such as frailty, patient preference, psychological and social needs; comorbidities are an important part of a holistic assessment. There are increasing pressures on MDTs in the UK, which mean that timely and accurate data are increasingly important, particularly in respect of cancer [8]. Two studies assessing MDT decision-making in an upper GI and CRC setting revealed that MDT decisions were not being implemented primarily due to a lack of information concerning patients’ comorbidities [9, 10]. Lack of relevant information on the burden of comorbidities and overall fitness of the patient may deter an MDT from making holistic decisions about the patient’s care and may necessitate repeated discussions at the MDT.

### ACE-27

The ACE-27 index was derived by Piccirillo and colleagues from the Modified Medical Comorbidity instrument which was itself derived from the Kaplan Feinstein Index (KFI). The ACE-27 is an example of organ/system-based approach; this methodology measures how comorbidity impacts on the functions of the bodily system, e.g. cardiovascular, gastrointestinal, respiratory etc. The aim of the ACE-27 development was to assess specifically for comorbidity in patients with cancer and has been utilised in a number of studies of which cancer was the context. Based on experience from research and clinical judgement, 27 conditions were identified and included into the index. The ACE-27 comorbidity index, similar to the KFI, grades the selected 27 comorbid conditions into 3 grades of severity from which they are summarised to give an overall rating. Specifically, comorbid diseases are classified separately as mild, moderate and severe in relation to the extent of organ decompensation and prognostic impact. These individual grades are then used to assign patients to the overall ACE-27 score, according to the highest ranked comorbidity. If patients have more than two moderate diseases in different organs an overall score of severe is assigned [3, 11]. It is one of several different comorbidity assessment tools and has been tested for validity and reliability in a number of studies; it can be completed by patients or by clinicians (see discussion below).

### Aims

In this study, we primarily aimed to determine the availability of comorbidity data in a CRC MDT and assessed the feasibility of routine comorbidity data collection using the validated ACE-27 questionnaire. Secondary aims of the study included determining the optimal time and method of comorbidity data collection.

## Methods

### Population

The study was conducted in an inner London CRC MDT serving a multi-ethnic, predominantly deprived population. The population was chosen given the comparatively high rate of colorectal cancer as against the UK average and the location of the research team. The London Borough of Tower Hamlets located in the eastern part of London has a population of 254,096, with one of the highest ethnic minority populations in the capital. Half of the borough’s population are from Asian or Asian British, Black or Black British or other ethnic background. Of these ethnic groups, Bangladeshis (32%) represent the largest ethnic group [12].

### Consent and ethics

The research team were drawn from the clinical team and had access to patient data. All patients gave appropriate consent for their data to be used in the study, which received relevant ethics approval.

### Context

We conducted this pilot study to assess availability of comorbidity data at CRC MDT and to find out the feasibility of using the validated ACE-27 questionnaire to capture this data. The NHS working party on comorbidity assessment in cancer report in 2001 recommended the use of the ACE-27 [13]. In 2010, after the 2009 national workshop on ‘Comorbidity in Cancer,’ the National Cancer Intelligence Network (NCIN) sought applications from MDT’s in England for pilot projects on collecting ACE-27 scores as a routine comorbidity measurement for use in MDT’s. The colorectal cancer MDT at The Royal London Hospital was recruited to evaluate the feasibility of collecting ACE-27 comorbidity scores in the context of CRC MDT.

### Phase I

In phase I, a retrospective mapping exercise was carried out during a 6-month period (January 2012 to June 2012) to assess the accuracy of the recorded patients’ comorbidities data during MDT meetings. Data pertaining to patients’ comorbidity status were collected from CRC MDT meeting records (historic cohort). Detailed evaluation of outpatient clinic letters, electronic patient records and general practitioner referral letters were carried out for all the patients in order to compare the completeness of comorbidity data available at the CRC MDT meeting and to delineate optimal times for data collection.

### Phase II

In phase II, comorbidity data were prospectively collected over a 3-month period using the validated ACE-27 comorbidity index. The ACE-27 was administered by the surgical team (consultant, specialty, core and foundation trainees) in surgical outpatient clinics, surgical ward and pre-assessment clinic to assess both the feasibility of routinely administering the ACE 27 forms and measuring comorbidity in CRC patients. This phase II firstly involved organising and appropriately training three foundation year 1 doctors as part of a team (juniors doctors within 1 year of qualifying) to conduct the ACE 27 questionnaire on 11 patients in two different settings. These settings were the (1) inpatient ward and (2) pre-assessment clinic.

### Review

A review of the process was then conducted following this period, to further train remaining members of the team and to resolve some of the discrepancies that occurred in the interpretation of the ACE-27 form and its use. Subsequently, the ACE-27 forms were completed prospectively in 50 colorectal patients.

## Results

### Phase I

**Fig. 1:**
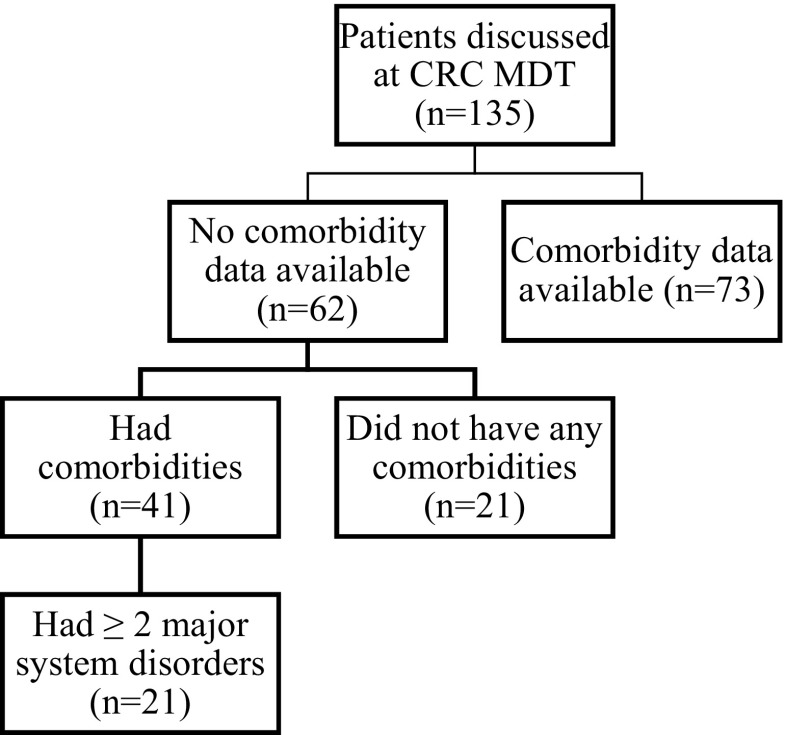
Flow chart summarising availability of comorbidity data at CRC MDT

In phase I, there were 135 patients who were newly referred to the CRC MDT meeting for first discussion. Of the 135 patients, 73 (54%) patients had comorbidity data available informing the CRC MDT meeting discussion. In the 62 patients (46%) with no comorbidity data available at MDT meeting, a detailed assessment of patient records revealed 41 (30%) patients to have comorbidities including ≥ 2 major system disorders in 21 (16%) patients (Fig. 1).

Of the 135 patients reviewed by the MDT, only 100 patients had available data to identify how quickly patients were discussed from time of referral to first discussion at the MDT meeting. This revealed that the majority (43%) of patients were discussed within 1 week of referral to the MDT meeting. However, the average lead time to discussion from referral to the CRC MDT meeting was 14 days (range 1 to 64 days). When the sources of referral for the 135 patients were mapped, it showed that common sources of patient referral to the MDT were surgical outpatient clinics (42%), emergency on-call take (18%) and the endoscopy (13%) department (Table 1).

**Table 1:** Sources of referral to MDT meetings

Source of referral	Number of patients
Emergency	24
Endoscopy	17
Surgical outpatients	57
In-hospital specialty ^A^	27
Tertiary	10

^A^General medicine, hepatopancreaticobiliary (HPB), urology etc.

### Phase II

**Fig. 2:**
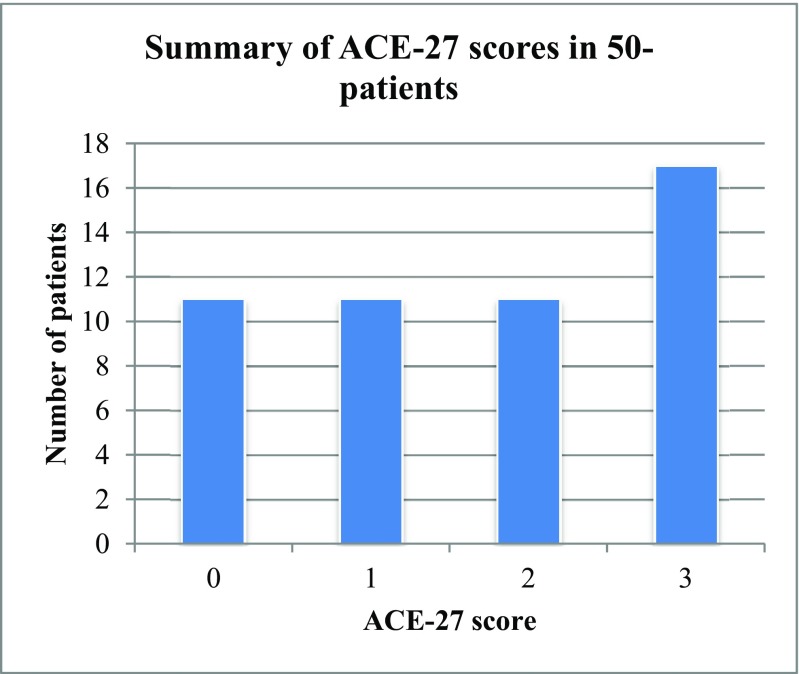
ACE-27 scores in 50-patients

In phase II, ACE-27 was administered in 50-patients in surgical outpatient clinics, surgical ward and pre-assessment clinic setting. The mean age was 54 years (range 20–84 years). Out of the 50 patients, 26 were male, while 24 were female. The average time to administer the ACE-27 was 4.8 min (range 1–15 min). In the grading of the ACE-27, 17 patients (34%) were grade 3, 11 patients (22%) were grade 2, 11 patients (22%) were grade 1 and 11 patients (22%) were grade 0 (Fig. 2).

## Findings

Phase I of the study found documented evidence of comorbidity data in 54% of patients only. Significantly, a further 16% of patients had comorbidities affecting ≥ 2 major systems. This knowledge would have been critical in the MDT’s decision-making. The study found that a structured approach of prospectively collecting comorbidity data, preferably using a validated questionnaire, would increase the availability of such data: in phase II, 100% of patients had comorbidity data. The ACE-27 questionnaire was easy to use, and produced consistent data for the MDT to interpret. As a result, the structured collation of comorbidity data has since been integrated into the MDT information process.

## Discussion

### Comorbidities

Comorbidities refer to diseases that coexist with the disease of interest. They are one of many patient data that are discussed at MDTs, such as patient preference, acceptability, frailty and psychological needs. The presence of comorbidities may alter treatment choice and correlates with survival and complications. The treatment of CRC can include extensive surgery, chemotherapy and radiotherapy; hence, such patients are, potentially, at higher risk of adverse outcomes. Decision-making at the MDT meeting is influenced by expertise, facility and resource factors, which would be similar for all patients discussed. However, the variable of patient factors changes with each patient discussed. Blazeby and colleagues assessed the reasons for discordance between the MDT decision and the final treatment implemented in an upper gastrointestinal cancer MDT setting. The majority (43.9%) were related to comorbid health issues [9]. Similarly, Wood and colleagues in a colorectal cancer MDT setting found the primary reason for an MDT decision not being implemented (40%) related to comorbid health issues [10].

### Cost benefits

MDT meetings use up significant resources and there are almost 1500 teams in England. The cancer MDT meetings are estimated to cost the NHS in England £50 million a year for preparation and a similar amount for attendance. Having real-time information about comorbidity would aid in making the MDT process more robust and decisive in optimising treatment, improving quality of survivorship and being cost-effective [8].

### Retrospective data collection

Phase I of the study confirmed the previously widely acknowledged view of poor comorbidity data availability within a CRC MDT discussion. Our study has shown on average there were 14 days from referral to discussion at the CRC MDT; hence, sufficient time exists to capture comorbidity data prior to discussion. Additionally, the mapping exercise revealed potential locations to administer the ACE-27 questionnaire, which includes surgical outpatient clinics, emergency department and at endoscopy.

### Prospective data collection

We initially planned to send the ACE-27 questionnaire to patients to fill out prior to their attending surgical outpatients or pre-assessment clinic; however, we judged that patients might struggle with the medical terminology, which precluded its use in patient questionnaires. Furthermore, this would have been inappropriate for our local population as English is not the first language for a significant majority. Instead, the ACE-27 was administered by junior and senior clinicians following a short 10-min training and on average took 4.8 min to complete prospectively. Training was embedded into the process of induction for all new members of staff as well as refresher training for existing members of staff; it was felt to be an extremely important and welcome part of discussing patients’ needs holistically. Roger et al.’s study used the ACE-27 index retrospectively and took on average of 10 min to complete [11]. They encountered difficulties in administering the ACE-27 retrospectively as many of the items listed in the index were not mentioned in medical notes and some of the mentioned comorbidities lacked sufficient detail about their severity to enable accurate scoring. Our study also found that using the ACE-27 index prospectively would be more feasible routinely rather than retrospectively.

### Future studies

Other comorbidity indices used in colorectal cancer reported in the literature include the Charlson comorbidity index (CCI), Elixhauser comorbidity index (ECI) and the National Institute on Ageing/National Cancer Institute Collaborative Study on Comorbidity and Cancer Index (NIA/NCI). CCI is an example of a weighted index, which consists of a number scoring system based on 19 weighted conditions that can subsequently be used for comparison and evaluation of risk. The ECI is an example of counts of individual disease and is a more recent risk-adjustment model comprising 31 conditions including weight loss and obesity. The NIA/NCI is another example of using counts of individual disease and was the only index designed exclusively utilising cancer patients in the NCI’s SEER program. The ELI and NIA/NCI evaluate the comorbidity burden only through a quantitative point of view, whereas the ACE-27 and CCI explore disease severity weighting the comorbid diseases on clinical impact. Studies in the literature found the aforementioned indices all have a good utility in predicting CRC patient’s survival [3, 14]. Future studies comparing the feasibility of collecting comorbidity data using the three indices against ACE 27 in CRC MDT setting are needed. We conducted our study in a specific national setting and local population; it would be valuable to test these findings in different settings and on a larger scale.

## Conclusions

Our study highlights the importance of having available a tool to collect comorbidity data prior to a CRC MDT discussion. Some limitations in the use of ACE-27 exist: different laboratory value units to those used in the UK and important comorbidities are omitted, which necessitate modifications [3, 11]. Further studies assessing the impact of ACE-27 comorbidity scores on the CRC MDT’s decision are required.
